# A Dual-Path Attention and Multi-Scale Fusion Network for Crop Disease and Pest Identification

**DOI:** 10.3390/e28091032

**Published:** 2026-09-20

**Authors:** Hong Zhang, Fagen Song, Yongqi Yuan, Ge Jin, Qian Zhang

**Affiliations:** 1 School of Information Engineering, Yancheng Institute of Technology, Yancheng 224051, China; zhanghong@ycit.edu.cn; 2 Jiangsu Provincial Engineering Technology Center for Multimodal Perception and Intelligent Control of Offshore Wind Power Systems, Yancheng 224051, China; 3 School of Information Technology, Jiangsu Open University, Nanjing 210036, China; yuanyq@jsou.edu.cn (Y.Y.);

**Keywords:** crop disease recognition, deep learning, attention mechanism, multi-scale feature fusion

## Abstract

Crop diseases and pests severely threaten global food security. While deep learning has shown promise in controlled settings, real-world field conditions—characterized by complex backgrounds, variable lesion scales, and high inter-class similarity—remain challenging. To overcome limitations in feature representation, scale adaptability, and model efficiency, we propose DPMFNet, a lightweight dual-path network integrating Spatial–Channel Dual-Attention (SCDA) and Multi-Scale Depthwise Separable Convolution (MDSC) modules. SCDA enhances critical regions via dynamic channel–spatial weighting with minimal overhead, while MDSC captures multi-scale contextual information through parallel dilated convolutions. Both are embedded into an improved residual block (AttMDSCBlock) to boost representational power while reducing parameters. A cross-attention mechanism fuses local details and global context from dual pathways, and a lightweight pyramid strategy adaptively integrates features across resolutions. Evaluated on PlantVillage and the AI Challenger 2018 dataset, DPMFNet achieves state-of-the-art accuracy among lightweight models, with only 14.24M parameters and 2.55G FLOPs. It demonstrates superior robustness in complex agricultural environments, balancing performance, efficiency, and deployability.

## 1. Introduction

Crop diseases and pests threaten global food production. Estimates suggest that 30% to 40% of annual yields are lost to plant pathogens, with developing countries hit hardest [1]. Traditional pest and disease identification mainly relies on the visual observation and experience of agricultural experts. This method is not only inefficient and subjective, but also difficult to meet the rapid diagnostic needs of large-scale planting areas. As computer vision and deep learning advanced, automated image-based recognition became a key direction in smart agriculture [2].

Convolutional neural networks (CNNs) have been widely used for this task due to their strong image modeling ability [3,4,5]. Early work applied models like AlexNet [6], VGGNet [7], and ResNet [8] to the PlantVillage dataset and reported over 95% accuracy [9]. But real fields are messy. Background clutter, changing light, and unpredictable lesion appearances reduce performance sharply [10]. In maize, for example, Northern Leaf Blight and Gray Leaf Spot look so alike that standard CNNs often confuse them [11].

Researchers have tried different fixes. Attention modules—SE [12], CBAM [13], Coordinate Attention [14]—help models focus on diseased areas by adjusting feature weights [15]. Multi-scale methods like FPN [16], ASPP [17], and Inception blocks [18] combine features from different layers to handle variation in spot size. Some also use GANs [19,20] to create synthetic images that mimic complex field backgrounds, making training data more diverse.

Recently, to overcome the limitations of classic off-the-shelf lightweight backbones (e.g., MobileNetV2, ShuffleNetV2) in complex agricultural scenes, several studies between 2023 and 2026 have tailored light-weight visual architectures specifically for plant pathology and pest identification. For instance, Duhan et al. [21] introduced LiteCShuffle, integrating a Channel Attention Module with channel shuffling inside inverted residual blocks to boost generalization across crops with minimal parameters. Hong et al. [22] proposed Light-HortiNet, combining a Mobile-Transformer backbone with a cross-scale lightweight attention branch to capture tiny pest targets under greenhouse conditions. Chen et al. [23] designed MoSViT, leveraging a localized precision attention mechanism within a compact Vision Transformer to capture multi-granularity symptom features. Sun et al. [24] presented an ultra-lightweight crop disease model utilizing efficient dual-dimension attention to resolve leaf occlusion in field environments. Furthermore, Zeng et al. [11] developed LDSNet, which combines dense multi-scale depthwise convolutions to handle highly variable lesion spot sizes.

Early work on crop disease recognition leaned heavily on classical image processing paired with machine learning classifiers. The pipeline usually split into two steps: handcrafting features, then feeding them to a classifier. Color histograms, texture descriptors like Local Binary Pattern (LBP), and shape metrics were common choices to encode symptoms. Brahimi et al. [25] though focused on deep learning for tomato diseases—also benchmarked these traditional features with SVMs, showing they could still perform well in controlled settings. Ebrahimi et al. [26] used SVMs on enhanced color and regional indices to detect thrips in strawberry canopies, keeping classification error below 2.25%. Wen et al. [27] built a local-feature system for orchard insect ID, while Wen and Guyer [28] later reported 86.6% accuracy on real field-collected insect images using a similar approach.

But these methods have clear drawbacks. Designing features by hand takes time—and expertise. Worse, the choices are subjective: one researcher’s “key texture” might be another’s noise. And they break down fast when images get blurry, lighting shifts, or weeds clutter the background. In short, they do not generalize beyond the lab. That fragility pushed the field toward deep learning, where features learn themselves from data—setting the stage for our end-to-end design.

Deep learning was a breakthrough. CNNs automatically build feature hierarchies from pixels, skipping manual engineering altogether. To boost performance in messy fields, researchers borrowed an idea from human vision: attention. By focusing only on relevant patches—like diseased spots—and ignoring distractions, models become more robust. Zeng and Li [29] plugged a self-attention block into a base CNN, calling it SACNN; it scored high on the MK-D2 dataset. Chen et al. [30] added a Location Soft Attention Module (LSAM) to MobileNetV2, forming Mobile-Atten. With two-stage transfer learning, they hit 98.48% accuracy on rice diseases—even with complex backgrounds.

At the same time, making models lightweight became urgent. Farmers do not run servers in their fields. Kamal et al. [31] redesigned depthwise separable convolutions for MobileNet, achieving 98.34% on PlantVillage with far fewer parameters. Waheed et al. [32] trimmed DenseNet for maize leaf disease and got 98.06% accuracy, cutting both model size and inference time.

These works inspired our design. Yet most use only one kind of attention—either spatial or channel-wise. That limits how well features can be refined across both dimensions. Our dual-path mechanism processes them in parallel, capturing richer diagnostic signals without bloating the network.

In real fields, disease symptoms come in all sizes. A spot might cover half a leaf—or hide in a corner. Distance, growth stage, and even camera angle change how big lesions can appear. This scale variability demands multi-scale reasoning. Zhang et al. [33] combined dilated convolutions with global pooling in GPDCNN for cucumber diseases, reaching 93.01% accuracy. Wang et al. [34] fused Inception blocks with dilated convolutions, expanding receptive fields to recognize 26 diseases across 14 crops at 99.18% accuracy.

Still, many fusion schemes stack scales one after another—or just run them side by side without real interaction. They miss chances to combine what each scale sees. Our pyramid module runs multiple scales in parallel and weights their contributions dynamically. The result? Better handling of size changes, whether a lesion is tiny or sprawling.

Still, problems remain. Many models cannot capture both fine details and the bigger picture at the same time. Their receptive fields are fixed—too small for large blights, too big for tiny spots. And high accuracy often comes with heavy models that will not run on a drone or smartphone in the field [35].

We propose DPMFNet, a Dual-Path Attention and Multi-Scale Fusion Network. It uses two parallel streams: one for local texture, one for global context. A cross-attention block lets them share useful signals. On top, a lightweight pyramid fuses features across scales, adapting to how big or small the lesions are. The main contributions of this work are as follows:

(1) A Spatial–Channel Dual-Attention (SCDA) module that merges average and max pooling to boost channel attention, paired with a spatial gate using dilated convolutions—highlighting key regions with almost no extra cost.
(2) A Multi-Scale Depthwise Separable Convolution group (MDSC), built with parallel depthwise layers at different dilation rates, embedded into a new residual unit (AttMDSCBlock). This cuts parameters and FLOPs while keeping rich feature diversity.
(3) Experiments using PlantVillage and AI Challenger 2018 show DPMFNet balances accuracy, size, and robustness well—making it suitable for real-time diagnosis in actual farming environments.

## 2. Materials and Methods

To enhance the capability of deep convolutional neural networks in plant disease and pest image recognition—specifically in terms of multi-scale feature perception, attention to diagnostically critical regions, and model lightweighting—we propose an improved ResNet34 architecture that integrates a lightweight Spatial–Channel Dual-Attention (SCDA) mechanism and a Multi-Scale Depthwise Separable Convolution Group (MDSC-Group), named DPMFNet. While preserving the original hierarchical structure of ResNet34, this model significantly boosts feature extraction performance through refined redesign of internal components within residual blocks, all while maintaining computational efficiency and compatibility with transfer learning.

### 2.1. Spatial–Channel Dual-Attention Mechanism (SCDA)

Although popular attention modules—such as Squeeze-and-Excitation (SE) [12], Convolutional Block Attention Module (CBAM) [13], Coordinate Attention (CA) [14], and recent agricultural attention variations —have been widely adopted, they present inherent structural limitations when applied to field crop images:

(1) Channel dimension: conventional modules like SE and CBAM heavily rely on Global Average Pooling (GAP) alone to compress global context, which tends to over-smooth localized, peak activation signals produced by small lesion spots or early-stage pests. In contrast, our SCDA module dynamically combines GAP with Global Max Pooling (GMP) via a parallel dual-path topology, retaining both overall symptom distribution and extreme regional features.
(2) Spatial dimension: traditional spatial attention branches (e.g., CBAM) utilize standard small 3×3 or 7×7 static convolutions, limiting their effective receptive field and failing to capture scattered, disjointed lesions across a single leaf. Recent light attention mechanisms (e.g., ECA or CA) capture 1D coordinate relationships but lack isotropic spatial context. Our SCDA incorporates dynamic dilated depthwise convolutions (r=2) inside the spatial gate, expanding the spatial receptive field to 7×7 without increasing network depth or depthwise parameters.
(3) Interaction topology: unlike sequential channel-then-spatial stacking (as in CBAM) which causes information bottleneck, SCDA performs parallel channel and spatial weight calculations, combining them via element-wise multiplication before adding back to the residual identity map. This ensures uninterrupted gradient flow and simultaneous spatio-channel reweighting tailored for field-captured disease spots.

Popular attention modules like SE and CBAM work well in many vision tasks—but they fall short when it comes to plant disease images. One issue is their reliance on Global Average Pooling (GAP) alone for channel weighting. GAP smooths everything out, which tends to miss small but critical spots. Another problem lies in spatial attention: most designs use small kernels, so they cannot see far enough to connect distant lesion parts.

**Figure 1:**
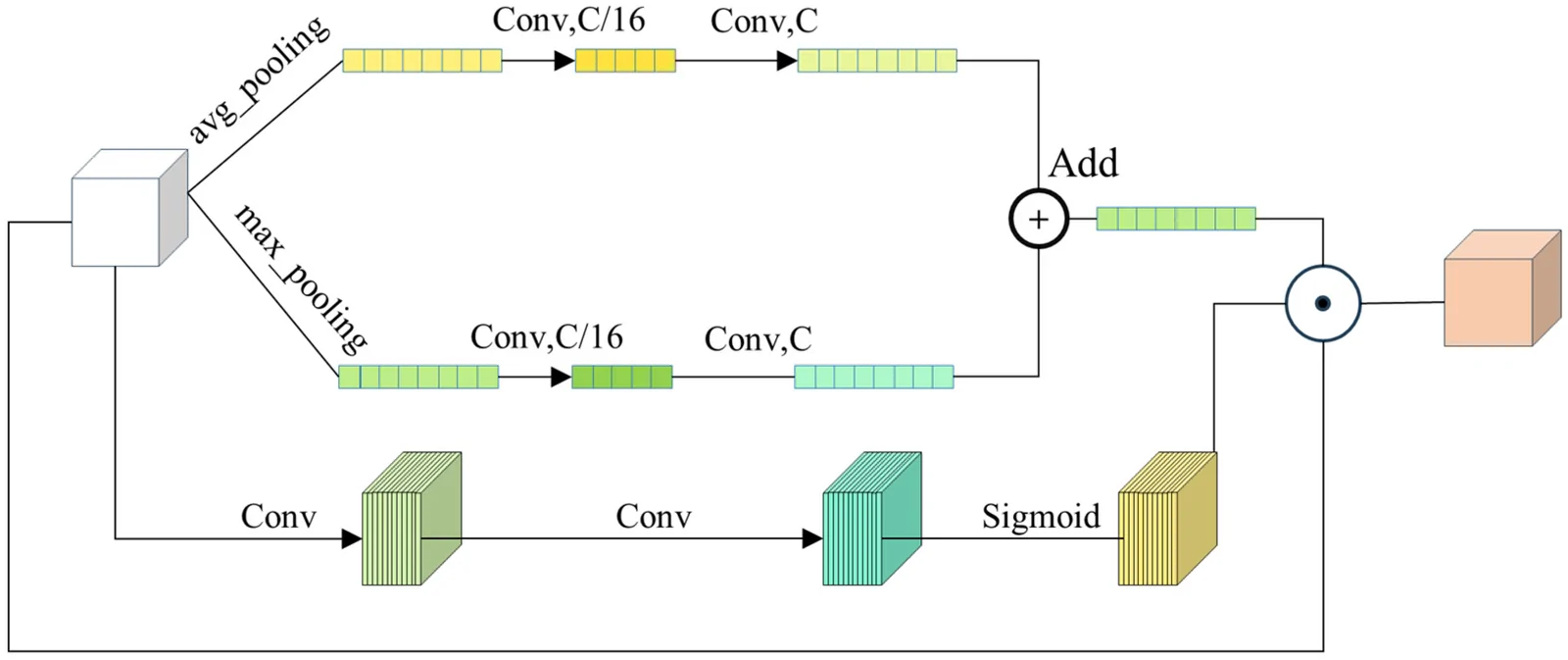
Schematic diagram of the SCDA network architecture.

To fix this, we introduce the Spatial–Channel Dual-Attention (SCDA) mechanism. It runs two paths in parallel—one for channels, one for space—and fuses them later for joint refinement. Given an input feature map $X\in {\mathbb{R}}^{C\times H\times W}$, SCDA processes both dimensions simultaneously and produces a reweighted version that better highlights diagnostic regions. The network structure diagram is shown in Figure 1.

#### 2.1.1. Dual-Path Channel Attention

Instead of using only GAP like the SE block, we combine it with Global Max Pooling (GMP). Why? Because GMP picks out the strongest activations—exactly where disease symptoms often appear. By running both pooling operations side by side, we capture not just the average response across a channel, but also its peak activity. This gives a richer signal for deciding which channels matter most. The features after pooling are defined as follows:

(1) $$Z_{\mathrm{avg}}=\frac{1}{H\cdot W}\sum_{i=1}^{H}\sum_{j=1}^{W}X(c,i,j),\;c=1,\ldots ,C$$

(2) $$Z_{\max}=\max_{i,j}X(c,i,j),\;c=1,\ldots ,C$$

Subsequently, the two pooled feature vectors are concatenated and passed through a shared two-layer fully connected structure (implemented via 1 × 1 convolutions) to reduce parameter count and enhance generalization capability:

(3) $$M_{c}^{\mathrm{avg}}\;=\;\sigma \left(W_{2}\cdot \mathrm{ReLU}\left(W_{1}\cdot Z_{\mathrm{avg}}\right)\right)$$

(4) $$M_{c}^{\max}\;=\;\sigma \left(W_{2}\cdot \mathrm{ReLU}\left(W_{1}\cdot Z_{\max}\right)\right)$$

where $W_{1}\in {\mathbb{R}}^{C/r\times C}$ and $W_{2}\in {\mathbb{R}}^{C\times C/r}$ are learnable parameters, r is the reduction ratio (set to 16 in this work), and σ(⋅) denotes the Sigmoid function. The final channel attention weights are given by the sum of the two paths:

(5) $$M_{c}\;=\;M_{c}^{\mathrm{avg}}\;+\;M_{c}^{\max}\in {\mathbb{R}}^{C\times 1\times 1}$$

This design enables the model to capture both global distribution trends and focus on the most salient activation channels, thereby enhancing sensitivity to lesion regions.

#### 2.1.2. Spatial Attention with Dynamic Receptive Field

Spatial attention is used to localize critical regions in the image. To expand the receptive field without increasing computational cost, we employ dilated convolution to construct the spatial attention branch:

(6) $$M_{s}\;=\;\sigma \left(\mathrm{Conv}\left(\mathrm{ReLU}\left(\mathrm{BN}\left(\mathrm{Conv}(X)\right)\right)\right)\right)\in {\mathbb{R}}^{1\times H\times W}$$

Here, the 1 × 1 convolution is used for dimensionality reduction (to lower computational cost), and the 3 × 3 dilated convolution with a dilation rate of 2 effectively expands the receptive field to 7 × 7 without increasing network depth through stacked convolutions. The resulting feature map is finally normalized via the Sigmoid function to obtain the spatial weight map.

#### 2.1.3. Dual-Attention Fusion Mechanism

In contrast to simple addition or concatenation, this work adopts an element-wise multiplication strategy to fuse channel and spatial attention, ensuring their synergistic interaction:

(7) $$Y\;=\;X\cdot \sigma (M_{c})\cdot M_{s}$$

This operation achieves dual optimization of “channel-wise selective enhancement + spatial precise localization,” which is particularly suitable for agricultural images with complex backgrounds and irregularly shaped lesions.

### 2.2. Multi-Scale Depthwise Separable Convolution Group (MDSC-Group)

#### Structural Innovation of Multi-Scale Depthwise Separable Group (MDSC)

Conventional multi-scale mechanisms, such as ASPP [17] and Inception blocks [18], capture multi-scale context by stacking standard convolutions with varying kernel sizes or dilation rates, leading to substantial computational overhead. MDSC differs in two critical ways:

(1) Parameter efficiency via multi-dilation depthwise convolutions: MDSC employs parallel 3 × 3 depthwise separable convolutions with dilation rates of r =1,2 and 3 (corresponding to effective receptive fields of 3×3, 7×7, and 11×11). This design handles wide lesion-scale variations while reducing computational FLOPs from $O(C\times C\times K^{2})$ to $O(C\times K^{2}+C\times C)$.
(2) Gradient-aware mean fusion vs. feature concatenation: Standard multi-scale architectures (e.g., FPN, ASPP) use channel concatenation, which rapidly increases channel dimensions and parameter counts in subsequent layers. MDSC employs a gradient-aware mean fusion strategy across parallel branches. This maintains constant channel dimensions, balances gradient propagation across all receptive scale branches during backpropagation, and prevents deep feature explosion.

**Figure 2:**
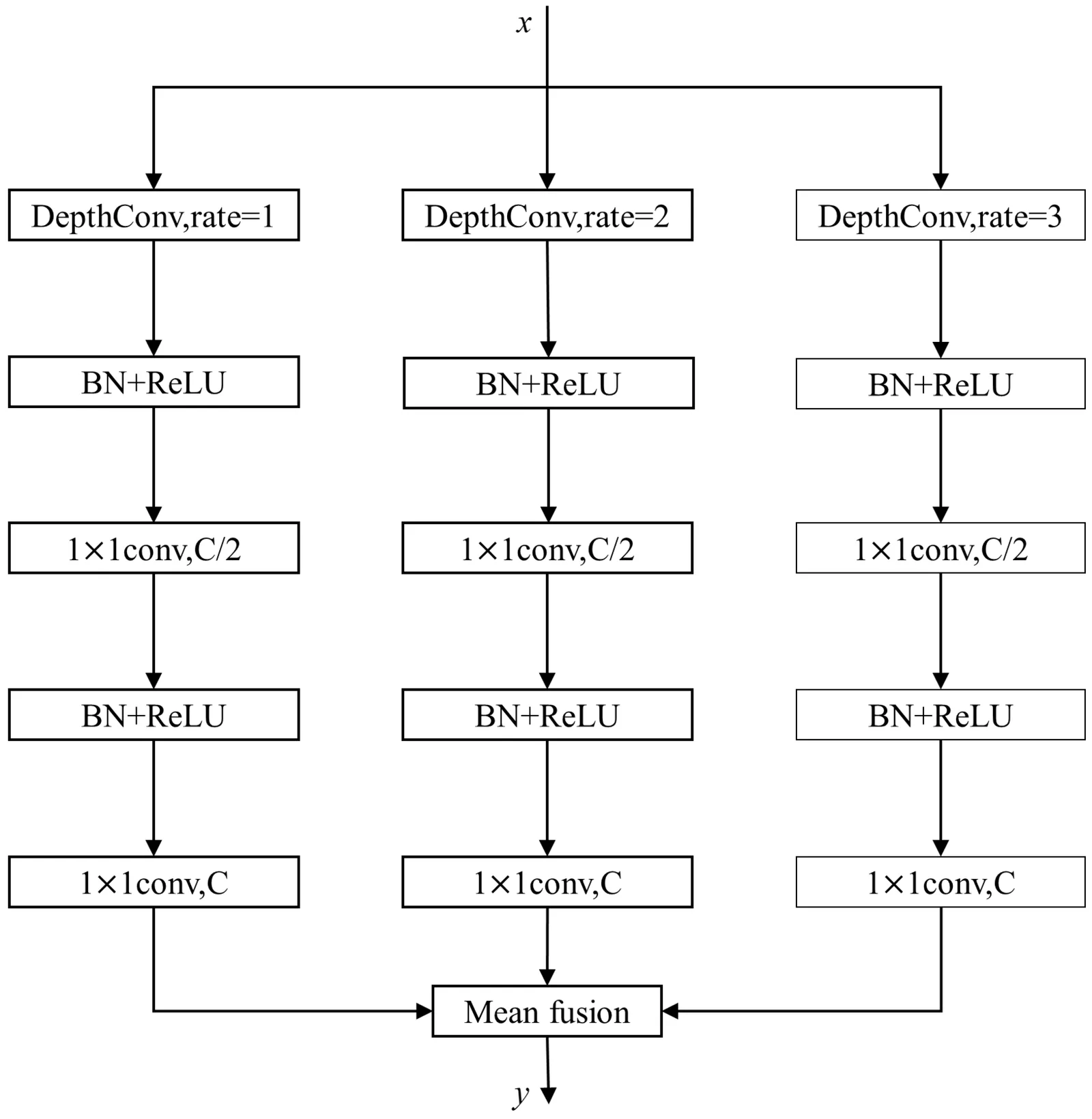
Schematic diagram of the MDSC-Group network architecture.

Standard convolution kernels have a fixed receptive field, making it difficult to adapt to lesions of varying sizes. While dilated convolutions can expand the receptive field, using a single dilation rate may lead to information omission. To address this issue, we design a Multi-Scale Depthwise Separable Convolution Group (MDSC-Group) that utilizes a parallel multi-branch architecture to achieve efficient multi-scale feature extraction. The network structure diagram is shown in Figure 2.

For an input $X\in {\mathbb{R}}^{C\times H\times W}$, the MDSC-Group consists of three parallel branches, each employing a 3 × 3 depthwise separable convolution with dilation rates d∈{1,2,3}. The structure of each branch is as follows:

Depthwise Convolution:

(8) $$X_{\mathrm{dw}}^{\left(d\right)}\;=\;{\mathrm{DepthConv}}_{d}(X)$$

The output remains with C channels.

Pointwise Convolution:

(9) $$X_{\mathrm{pw}1}^{\left(d\right)}\;=\;\mathrm{ReLU}\left(\mathrm{BN}\left({\mathrm{Conv}}_{1\times 1}(X_{\mathrm{dw}}^{\left(d\right)})\right)\right)$$

The number of output channels is C/2.

Pointwise convolution to restore the number of channels:

(10) $$X_{\mathrm{out}}^{\left(d\right)}=\mathrm{BN}\left({\mathrm{Conv}}_{1\times 1}(X_{\mathrm{pw}1}^{\left(d\right)})\right)$$

The channel dimension is then restored to C.

The outputs of the three branches are fused using a gradient-aware averaging fusion strategy:

(11) $$Y\;=\;\frac{1}{3}\sum_{d=1}^{3}X_{\mathrm{out}}^{\left(d\right)}$$

The spatial branch goes further. It uses dilated convolutions at multiple rates to expand what the model “sees.” Kernels with dilation 1, 2, and 3 effectively cover 3 × 3, 7 × 7, and 11 × 11 regions—ideal for lesions that range from tiny specks to large blights. And thanks to depthwise separable convolutions, the cost stays low: computational load drops from $K^{2}\cdot C_{\mathrm{in}}\cdot C_{\mathrm{out}}$ to $K^{2}\cdot C_{\mathrm{in}}+C_{\mathrm{in}}\cdot C_{\mathrm{out}}$, cutting FLOPs significantly.

We also avoid concatenating the branches. Instead, we average their outputs. This keeps gradients flowing cleanly through each path during training without blowing up the parameter count. The result is stable learning—and features that stay sensitive to both fine details and broader patterns.

### 2.3. Enhanced Residual Block (AttMDSCBlock)

**Figure 3:**
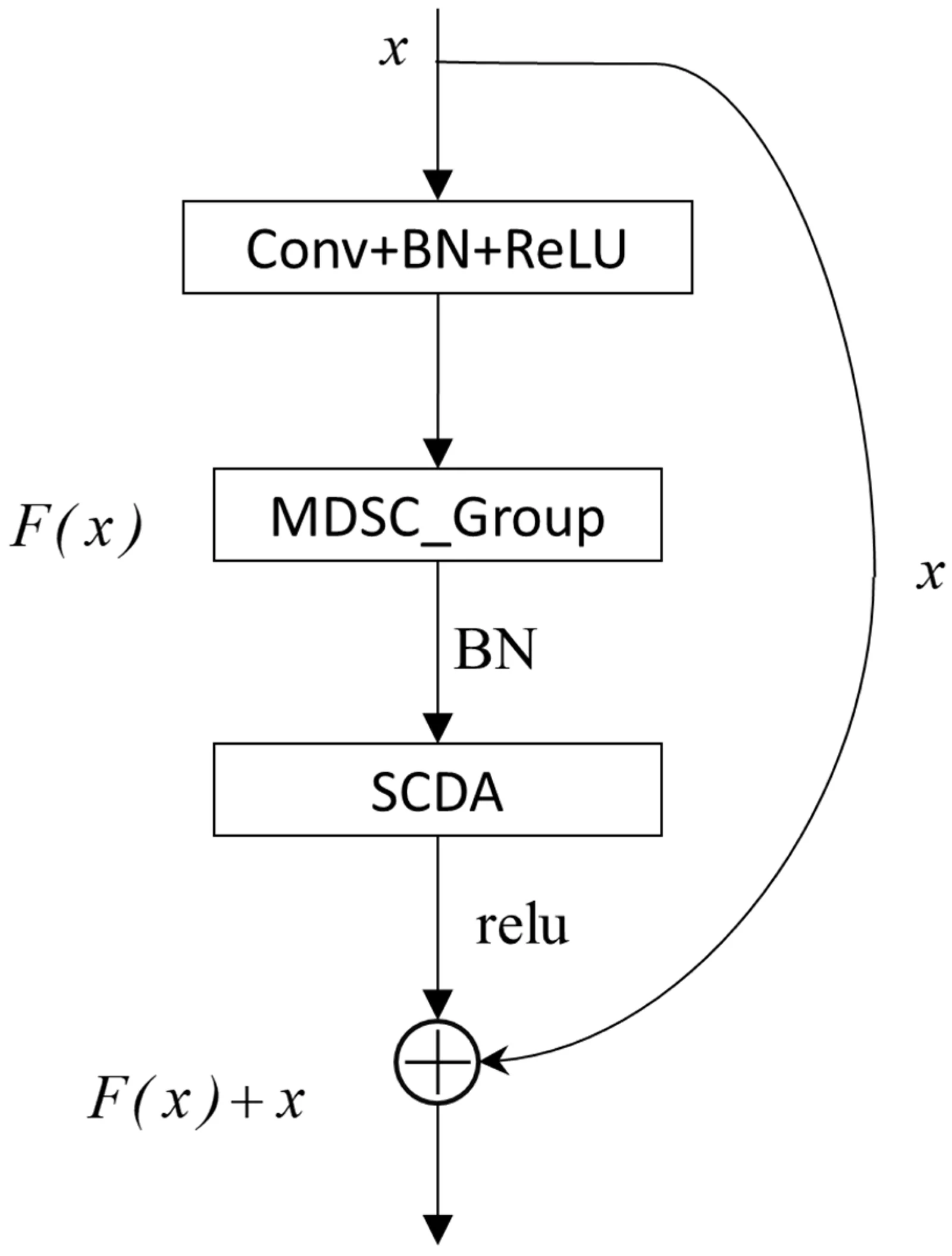
Schematic diagram of the Enhanced Residual Block (AttMDSCBlock).

We build our core unit on top of the classic ResNet block, calling it AttMDSCBlock (see Figure 3). Three key changes make it better suited for disease recognition:

First, we swap out the second 3 × 3 convolution for our MDSC-Group. This brings multi-scale context into the residual stream—helping the network handle lesions of any size.

Second, we plug the SCDA module right before the final addition in the residual path. That way, the attention-enhanced features get merged with the original shortcut signal.

Third—and importantly—we place SCDA before the skip connection is added. This ensures the identity mapping stays untouched. Only the transformed branch gets reweighted. In practice, this prevents attention from affecting gradient flow, which makes training more stable, especially on small datasets.

The forward pass follows the standard residual pattern, with these upgrades layered in at precise points to maximize impact without adding bulk.

### 2.4. Overall Network Architecture

Our DPMFNet sticks closely to the ResNet34 backbone. It has four stages—conv2_x through conv5_x—with 3, 4, 6, and 3 blocks respectively, totaling 34 layers. A comparison between the original ResNet34 and our modified version is provided in Table 1.

At the end, a standard classification head takes over: Global Average Pooling → flatten → fully connected layer → output logits for N classes. This design keeps compatibility with pretrained models, easing transfer learning.

We start by loading official ResNet34 weights. Since our changes begin at conv3_x (where MDSC-Group and SCDA are inserted), only early layers—conv1, bn1, layer1, and part of layer2—share parameters with the original. During implementation, we match keys in the state_dict: compatible weights are loaded directly; everything else is randomly initialized.

To protect low-level features—like edges and textures—that are useful across tasks, we allow selective freezing. For example, conv1, bn1, and layer1 can be frozen during early fine-tuning. Only higher layers adapt to the new disease dataset. Formally, the freezing strategy is expressed as

(12) $$H_{1}\;=\;\mathrm{ReLU}\left(\mathrm{BN}({\mathrm{Conv}}_{3\times 3}(X))\right)$$

(13) $$H_{2}\;=\;\mathrm{BN}\left(\mathrm{MDSC}-\mathrm{Group}(H_{1})\right)$$

(14) $$H_{2^{\prime }}\;=\;\mathrm{SCDA}(H_{2})$$

(15) $$F(X)=\left\{\begin{array}{ll} X, & \mathrm{if}\;\mathrm{stride}=1\;\mathrm{and}\;C_{\mathrm{in}}=C_{\mathrm{out}}\\ {\mathrm{Conv}}_{1\times 1}(X), & \mathrm{otherwise} \end{array}\right.$$

(16) $$Y=\mathrm{ReLU}\left(H_{2^{\prime }}+F(X)\right)$$

This approach strikes a balance: it reuses general visual knowledge while leaving room for task-specific learning. This is crucial in agriculture, where labeled field images are often limited.

## 3. Results

### 3.1. Datasets

Our experiments are conducted on two widely used public plant disease image datasets: AI Challenger 2018 and PlantVillage. The AI Challenger 2018 dataset, released by organizations including Sinovation Ventures, focuses on fine-grained crop disease recognition under real-world field conditions. It covers 10 major crops—such as apple, corn, grape, rice, potato, and tomato—and includes 27 distinct disease types. For 24 of these diseases, symptom severity is further categorized into “mild” and “severe,” resulting in a total of 61 fine-grained classes when combined with the healthy category. The dataset contains 36,224 RGB images, split into 28,517 for training, 3203 for validation, and 4504 for testing. Images exhibit complex backgrounds (e.g., soil, weeds, occlusions), uneven illumination, and highly variable lesion scales, making it a challenging benchmark for evaluating model robustness in practical agricultural scenarios. In contrast, the PlantVillage dataset used in this work is obtained from the Baidu AI Studio platform (https://aistudio.baidu.com/datasetdetail/245434 (accessed on 28 June 2026)), which provides an augmented version of the original PlantVillage dataset. This version contains 60,343 high-quality RGB images spanning 38 classes (37 diseases plus one healthy class) across 14 crop species. All images in this version are captured against a uniform white background under controlled lighting, yielding clear lesion regions with minimal visual interference. We adopt an 80/10/10 split for training, validation, and testing, resulting in 48,265 training images, 6034 validation images, and 6044 test images. By evaluating on both datasets—one representing idealized laboratory conditions and the other reflecting messy field environments—we comprehensively assess the generalization capability, fine-grained discrimination, and real-world applicability of the proposed DPMFNet model.

### 3.2. Experimental Details

All experiments are implemented using PyTorch 2.4.1 with torchvision 0.19.1 on a Windows system equipped with an Intel(R) Core(TM) i9-14900K CPU (3.20 GHz), 128 GB RAM, and an NVIDIA GeForce RTX 4090 GPU (24 GB VRAM), supported by Python 3.8 and CUDA Toolkit 12.1. To ensure fairness and reproducibility, all baseline models—including AlexNet, VGG16, ResNet18, ResNet34, ResNet50, MobileNetV2, DenseNet121, GoogLeNet, ShuffleNet_v2_x0_5, ShuffleNet_v2_x1_0, ConvNeXt-T, and ConvNeXt-S—are directly adopted from the official torchvision.models implementations without any architectural modifications. Our DPMFNet is constructed by replacing specific components in the original ResNet34 as detailed in Section 3. All models are initialized with ImageNet-pretrained weights to benefit from effective parameter initialization.

For data preprocessing, all input images are resized to 224 × 224 pixels using bilinear interpolation. During training, we apply random horizontal flipping with a probability of 0.5 and random rotation within ±15 degrees as the primary augmentation strategies. No additional color jittering or random cropping is employed, so as to preserve the original lesion morphology and avoid distorting diagnostic texture cues. Both training and validation images are normalized using the ImageNet channel-wise statistics (mean = [0.485, 0.456, 0.406], std = [0.229, 0.224, 0.225]). The validation set is evaluated solely with resizing and normalization, without any stochastic augmentation, ensuring consistent and reproducible evaluation across all models.

During training, we uniformly employ the Adam optimizer with an initial learning rate of 0.001, β_1_ = 0.9, β_2_ = 0.999, and weight decay of 1 × 10^−4^. The learning rate is dynamically adjusted by a ReduceLROnPlateau scheduler that monitors validation loss, with a reduction factor of 0.3, a patience of 5 epochs, and a minimum learning rate of 1 × 10^−6^. The batch size is set to 128 for both training and validation. Training proceeds for up to 200 epochs, but an early stopping mechanism halts the process if validation accuracy fails to improve for 50 consecutive epochs. To guarantee reproducibility, the global random seed for NumPy, PyTorch, and Python’s random module is fixed at 2022. Input images are resized to 224 × 224 pixels and augmented using random horizontal flipping, random rotation within ±15 degrees, and normalization based on ImageNet statistics (mean: [0.485, 0.456, 0.406], std: [0.229, 0.224, 0.225]).

The loss function is the standard cross-entropy loss, defined as

(17) $$L\;=\;\frac{1}{N}\sum_{i}L_{i}\;=-\frac{1}{N}\sum_{i}\sum_{c=1}^{M}y_{ic}\log\left(p_{ic}\right)$$

where M denotes the number of classification categories; $y_{\mathrm{ic}}$ is the indicator function, which equals 1 if the true label of the image is class c and 0 otherwise; and $p_{ic}$ represents the predicted probability that image i belongs to class c.

### 3.3. Evaluation Metrics

To evaluate model performance, we adopt four standard metrics: accuracy, precision, recall, and F1-score. These are computed based on the confusion matrix counts

(18) $$\;\mathrm{Accuracy}=\frac{TP+TN}{TP+TN+FP+FN}$$

(19) $$\;\mathrm{Precision}=\frac{TP}{TP+FP}$$

(20) $$\mathrm{Recall}\;=\;\frac{TP}{TP+FN}$$

(21) $$\;F1\;\mathrm{Score}=2\;\cdot \;\frac{\mathrm{Precision}\cdot \mathrm{Recall}}{\mathrm{Precision}+\mathrm{Recall}}$$

where TP (True Positives) refers to correctly predicted positive samples, TN (True Negatives) to correctly predicted negative samples, FP (False Positives) to negative samples incorrectly classified as positive, and FN (False Negatives) to positive samples misclassified as negative. The metrics provide a balanced assessment of classification quality, particularly important in the presence of class imbalance commonly found in agricultural disease datasets.

Accuracy is reported as the overall accuracy (the proportion of correctly classified samples among all samples). Precision and F1-score are computed as weighted averages, where the metric for each class is weighted by the number of samples in that class. Recall is computed as macro-average, where the metric is first calculated independently for each class and then averaged across all classes. We adopt this mixed evaluation protocol because it aligns with common practice in multi-class classification benchmarks and provides a comprehensive assessment of model performance: weighted averages reflect performance on majority classes, while macro-average recall ensures that minority classes are not neglected in the evaluation.

### 3.4. Experimental Results and Analysis

To account for the inherent variability of the test set, we report all quantitative metrics in Table 2 and Table 3 as mean ± standard deviation derived from a Bootstrap resampling procedure with 1000 iterations on the fixed test splits. Since both datasets provide official train/validation/test partitions and we fix the random seed for reproducibility, repeated independent training runs are not feasible; Bootstrap offers a statistically sound way to estimate the uncertainty of our evaluation. Specifically, for each iteration, we randomly draw with replacement a subset of the test set of the same size as the original test set, compute all evaluation metrics (accuracy, precision, recall, and F1-score) on this resampled subset, and record the results. The reported mean and standard deviation are then calculated across the 1000 Bootstrap samples. All metrics are expressed in percentage points (%), and the standard deviation thus carries the same unit (percentage points). This procedure provides a robust estimate of the sampling variability of our evaluation metrics without requiring multiple expensive training runs.

**Figure 4:**
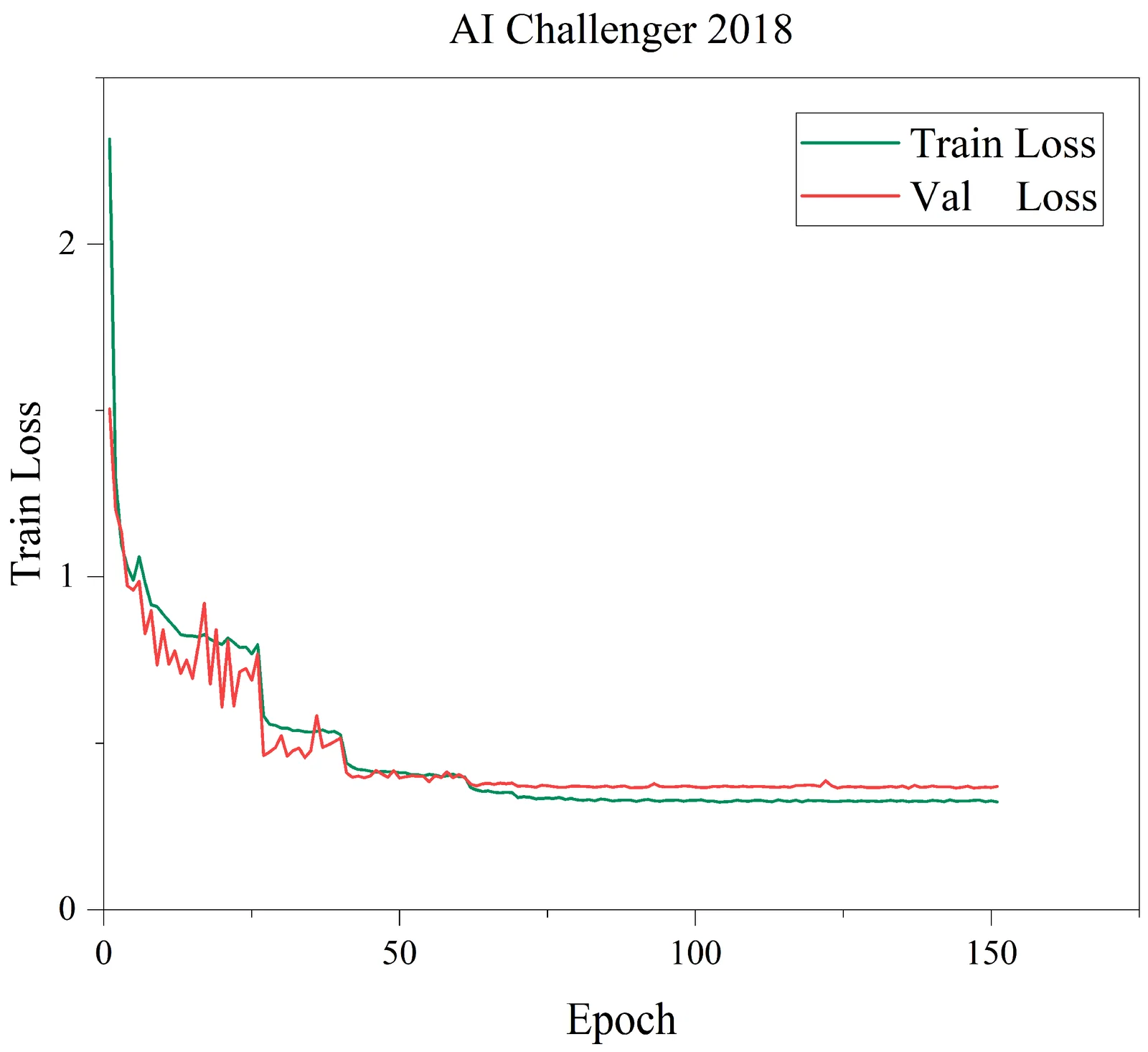
Training and validation loss curves of DPMFNet on the AI Challenger 2018 dataset.

**Figure 5:**
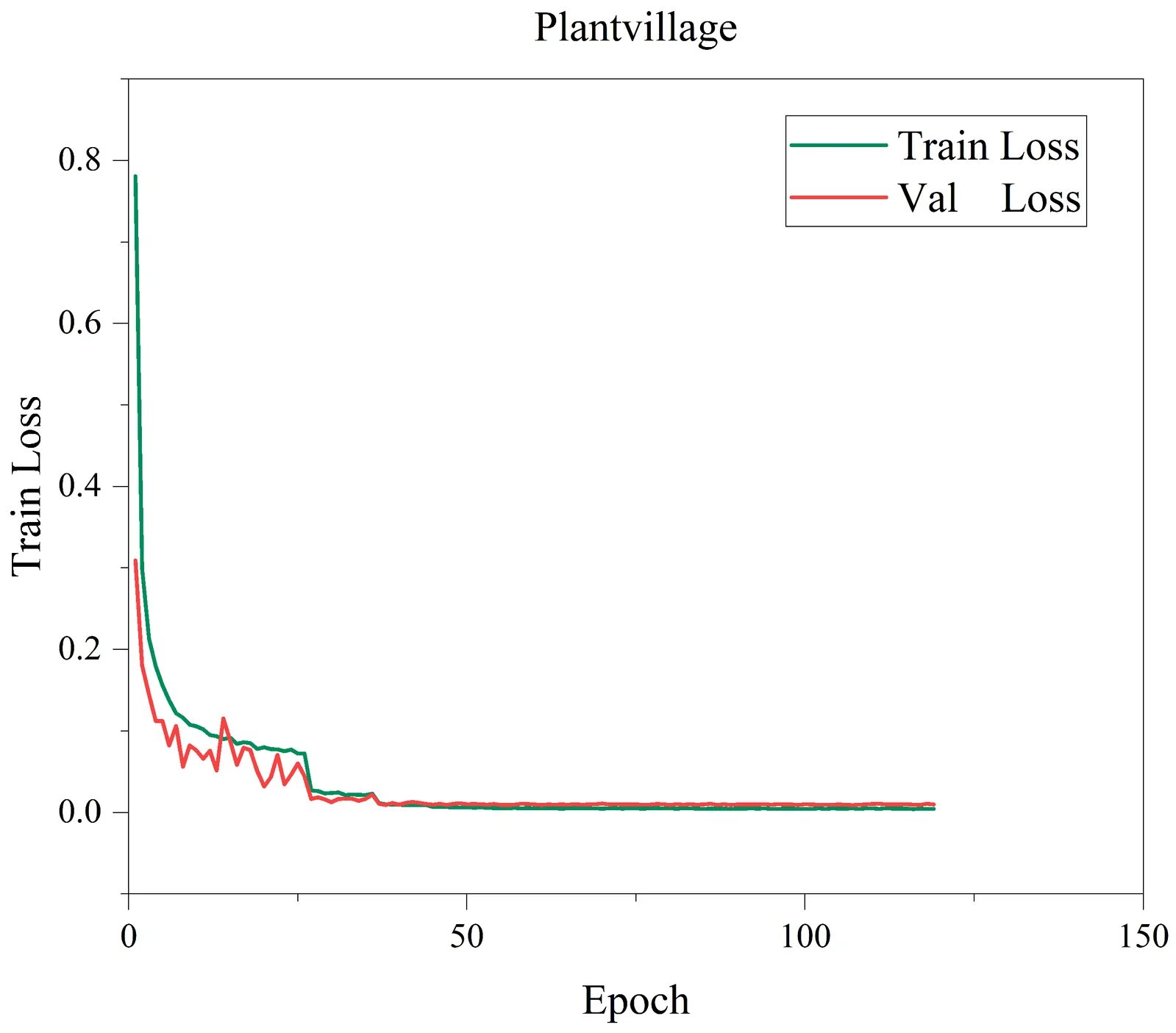
Training and validation loss curves of DPMFNet on the PlantVillage dataset.

**Figure 6:**
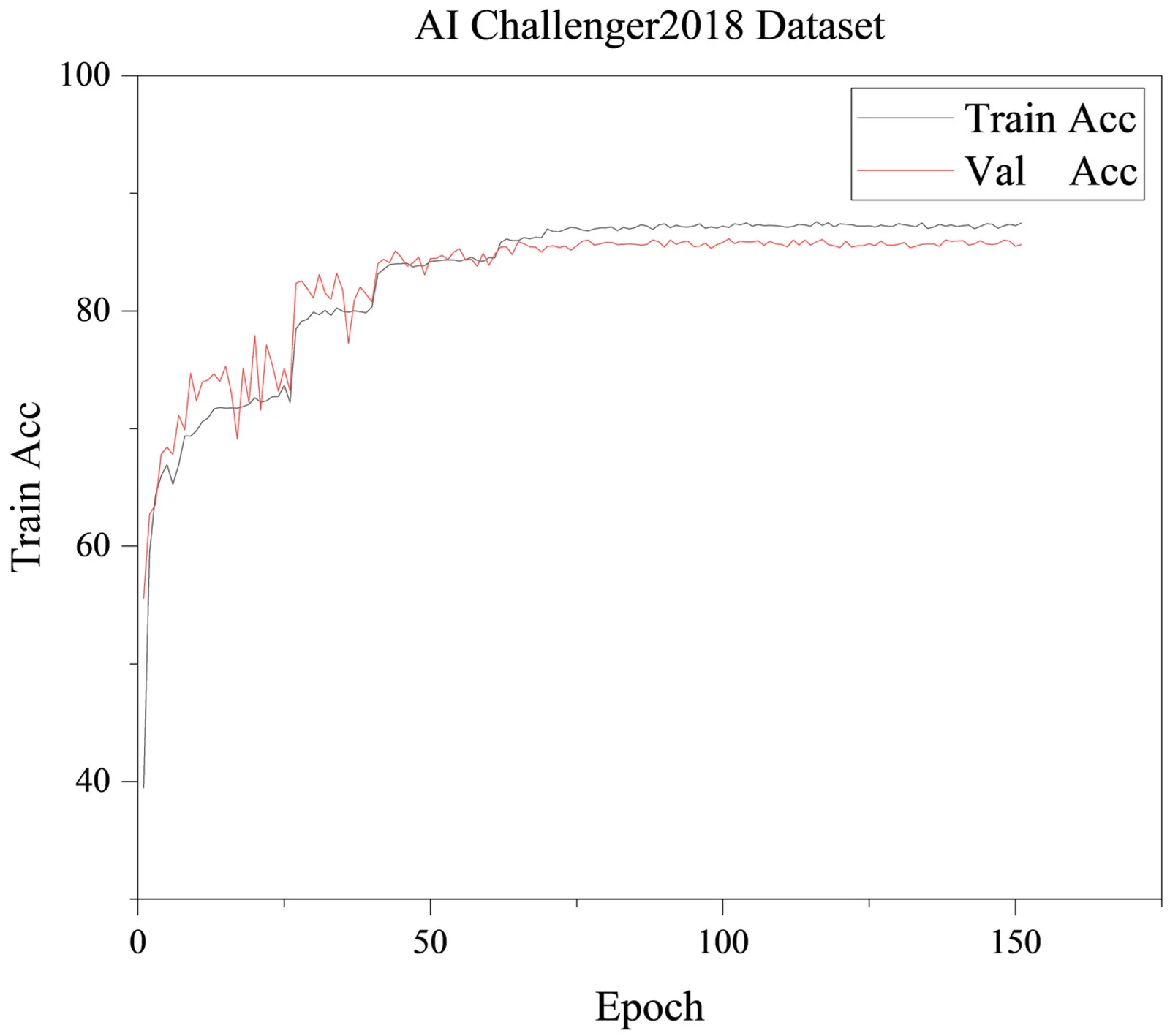
Training and validation accuracy curves of DPMFNet on the AI Challenger 2018 dataset.

**Figure 7:**
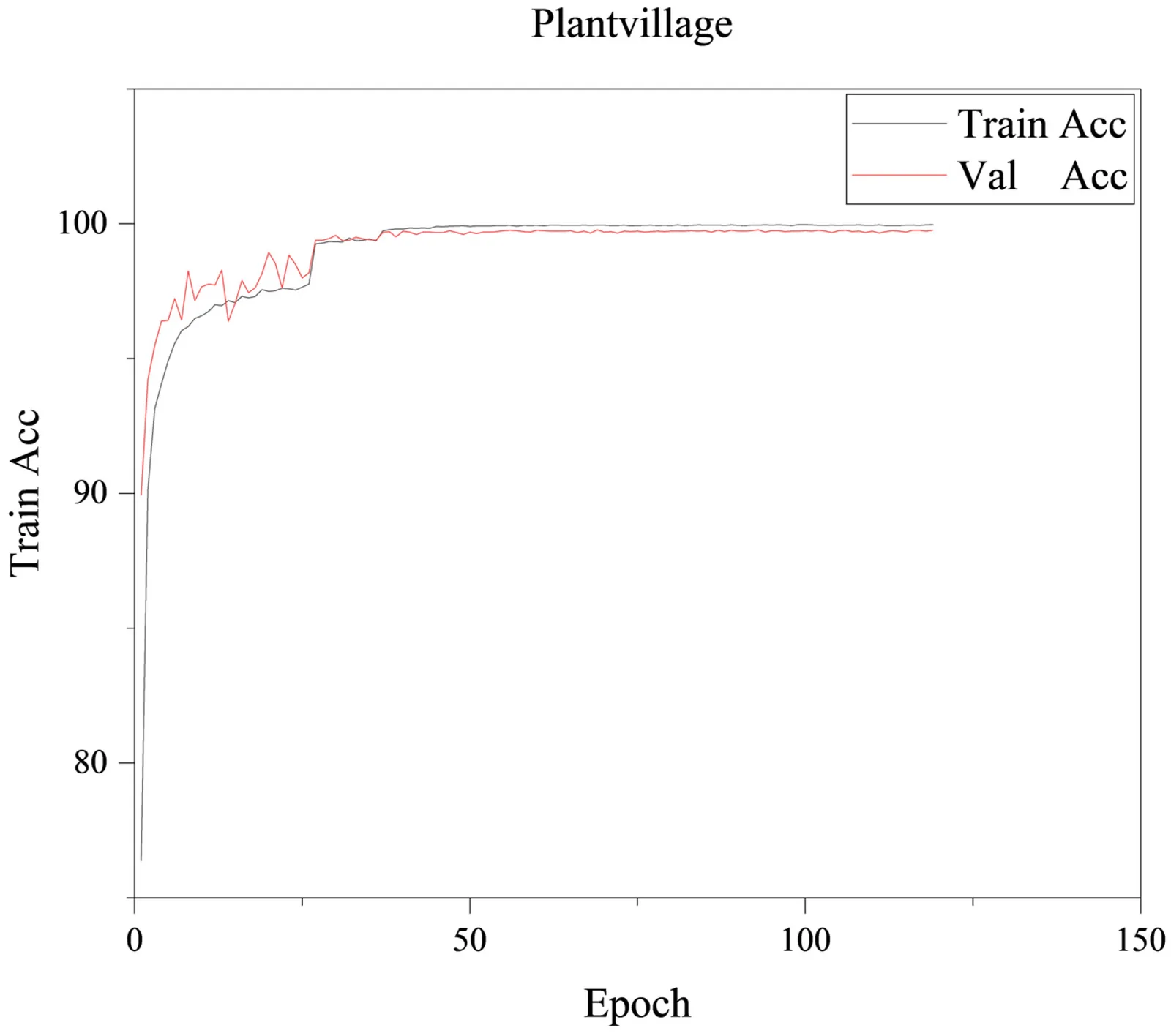
Training and validation accuracy curves of DPMFNet on the PlantVillage dataset.

We evaluate DPMFNet against 16 strong baselines—including both CNNs and Vision Transformers—on two widely used plant disease datasets: PlantVillage (clean, lab-style images) and AI Challenger 2018 (real-field, highly variable conditions). Training and validation loss curves are shown in Figure 4 and Figure 5, accuracy trends in Figure 6 and Figure 7, and final quantitative scores summarized in Table 2 and Table 3.

On PlantVillage, the model converges fast: both training and validation loss drop close to zero within about 50 epochs, with the two curves nearly overlapping throughout—suggesting rapid learning of confident features and almost no overfitting. Accuracy tells a similar story: Train Acc and Val Acc both climb above 99.6% by epoch 50 and stay within 0.3% of each other for the rest of training. That kind of stability makes sense given how uniform this dataset is—centered leaves, plain backgrounds, consistent lighting.

The real test comes with AI Challenger 2018, where things become more complicated. Initial loss starts high (>2.0), and early accuracy hovers below 20%, reflecting the challenge of class imbalance, cluttered backgrounds, and blurry lesions. But, by around epoch 70, the model settles down: final training accuracy reaches ~87%, validation ~86.5%, with a gap under 0.5%. There is some variation in Val Acc between epochs 50–70, likely due to noisy samples, but the overall trajectory keeps rising. Crucially, the loss and accuracy curves never diverge, which tells us the model is not memorizing; it is generalizing.

This efficient convergence (well before the 200-epoch cap) is partly thanks to our early stopping rule (patience = 50), which halts training once validation accuracy stops improving. But the architecture itself plays a bigger role. The SCDA module helps gradients flow more effectively by dynamically reweighting spatial and channel features, so the network zeroes in on real symptoms faster. Meanwhile, the MDSC-Group pulls in multi-scale context in one go—no need to wait dozens of extra epochs for the model to “figure out” scale variations. Together, they let us hit high performance without burning through compute.

Looking at the numbers in Table 2 and Table 3, DPMFNet achieves 86.48% ± 0.50% accuracy on AI Challenger 2018—beating the next best method (ShuffleNetV2_x1_0 at 85.28% ± 0.51%) by over 1.2 points. It also leads in precision (86.63% ± 0.50%), recall (86.48% ± 0.50%), and F1-score (86.33% ± 0.51%), showing that it does not just get the easy cases right but handles hard, ambiguous ones too.

Even on the “easy” PlantVillage set, DPMFNet still edges out top performers: 99.72% ± 0.07% accuracy vs. DenseNet121 (99.67% ± 0.07%) and ConvNeXt-B (99.52% ± 0.08%), with an F1-score matching its accuracy at 99.72% ± 0.07%. This is not overfitting—it is better feature discrimination, enabled by the joint action of attention and multi-scale fusion.

Finally, the model stays lean and exhibits favorable runtime characteristics for real-world deployment. As shown in Table 4, at just 14.24 million parameters and 2.55G FLOPs, it is notably smaller than ResNet34 (21.3M, 3.68G) and competitive with most lightweight designs—only losing to ultra-compact models like EfficientNet-B0 (4.06M) or ShuffleNetV2_x1_0 (1.29M), which sacrifice accuracy for size. Beyond theoretical complexity, we further evaluate practical deployment metrics on an NVIDIA RTX 4090 GPU. The measurement protocol is as follows: inference batch size is 128, precision is FP32, with two warm-up batches and all subsequent batches measured for latency; CUDA events and synchronization are used for accurate timing, and only the forward pass is timed (preprocessing and data transfer are excluded). DPMFNet achieves an inference latency of 0.74 ms and a throughput of 1350.03 FPS. While this is slightly higher than ResNet34 (0.35 ms, 2878.16 FPS) and VGG16 (0.69 ms, 1441.78 FPS), it is substantially lower than heavyweight Vision Transformers such as ConvNeXt-T (0.86 ms, 1156.11 FPS) and ConvNeXt-S (1.48 ms, 675.15 FPS), and remains competitive with ResNet50 (0.61 ms, 1627.78 FPS) given the large accuracy gain. More importantly, the peak GPU memory consumption of DPMFNet is only 921.45 MB, which is favorably below ResNet34 (948.06 MB), far less than ResNet50 (1446.54 MB) and ConvNeXt-T (1660.97 MB), and even comparable to the much shallower ResNet18 (909.46 MB). These results confirm that our efficiency gains—replacing standard convolutions with depthwise separable ones via the MDSC-Group, and computing SCDA attention through shared FC layers and dilated convolutions—effectively translate into not only low theoretical FLOPs and parameters but also hardware-friendly memory footprints and practical inference speed. This combination demonstrates that DPMFNet exhibits favorable efficiency characteristics on GPU platforms. Actual on-device validation on embedded hardware is left as an important future direction.

### 3.5. Qualitative Visualization Analysis

To provide intuitive evidence for the feature-learning behavior of our proposed DPMFNet and to validate its effectiveness in crop disease recognition, we conduct qualitative visual analyses on the PlantVillage dataset.

**Figure 8:**
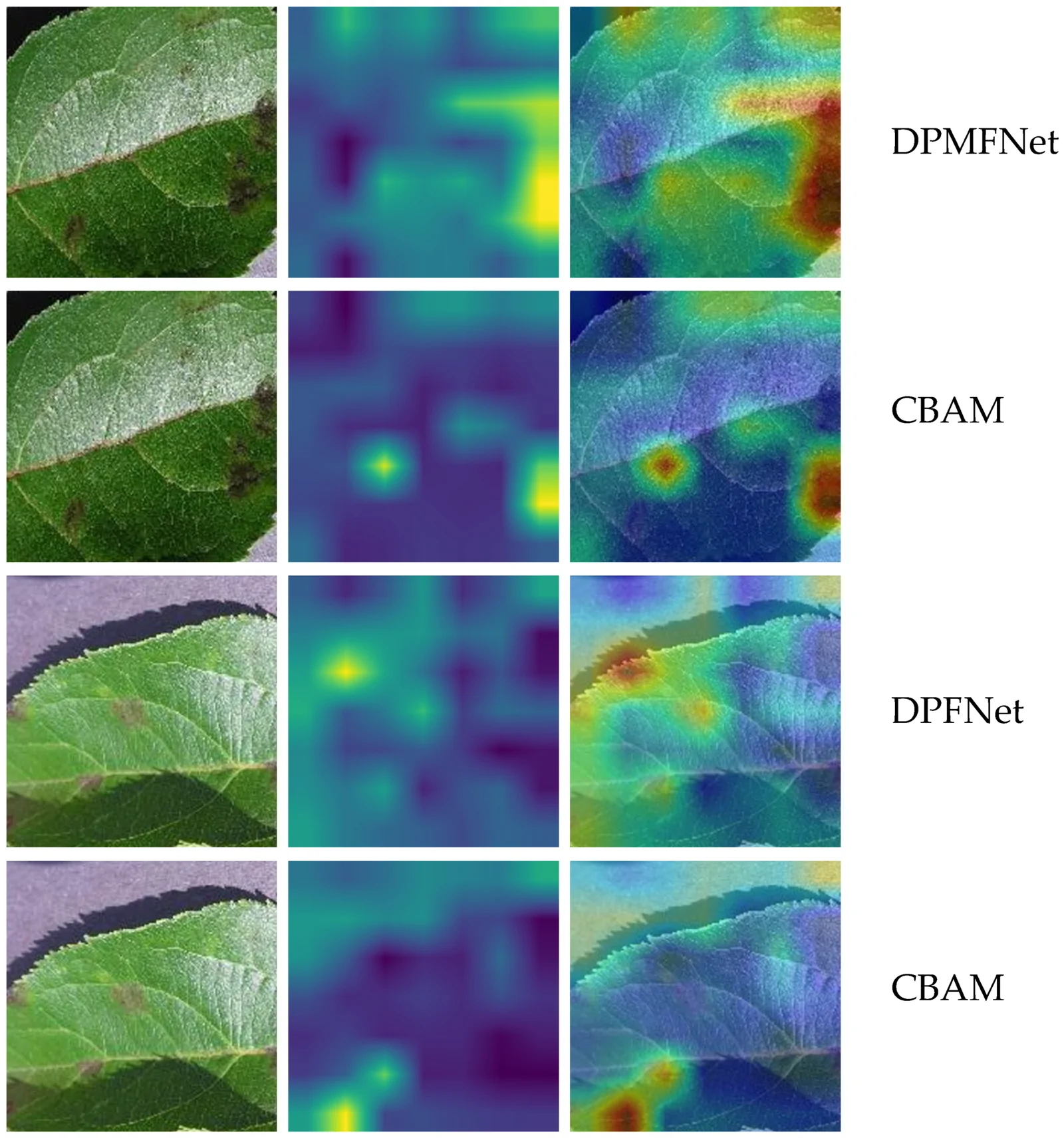
Grad-CAM activation map comparison: CBAM vs. our SCDA module.

Class activation mapping comparison: We employ Grad-CAM to visualize the discriminative regions highlighted by our SCDA module, using the classic Convolutional Block Attention Module (CBAM) embedded in the same ResNet34 backbone as the baseline for comparison. As shown in Figure 8, CBAM produces activation maps that, while generally focusing on the leaf regions, often exhibit scattered and less concentrated responses, with some attention spilling over to healthy leaf margins or background boundaries. In contrast, our SCDA module generates more compact and precisely localized activation maps that tightly adhere to the actual lesion boundaries. This improvement stems from two key designs: (1) the parallel fusion of Global Average Pooling and Global Max Pooling in the channel branch preserves both overall symptom distribution and extreme peak responses, and (2) the dynamic dilated depthwise convolution in the spatial branch expands the effective receptive field to capture lesion-related spatial context more effectively. Consequently, SCDA achieves more focused attention on true symptomatic regions, confirming its advantage over conventional sequential channel–spatial attention mechanisms.

**Figure 9:**
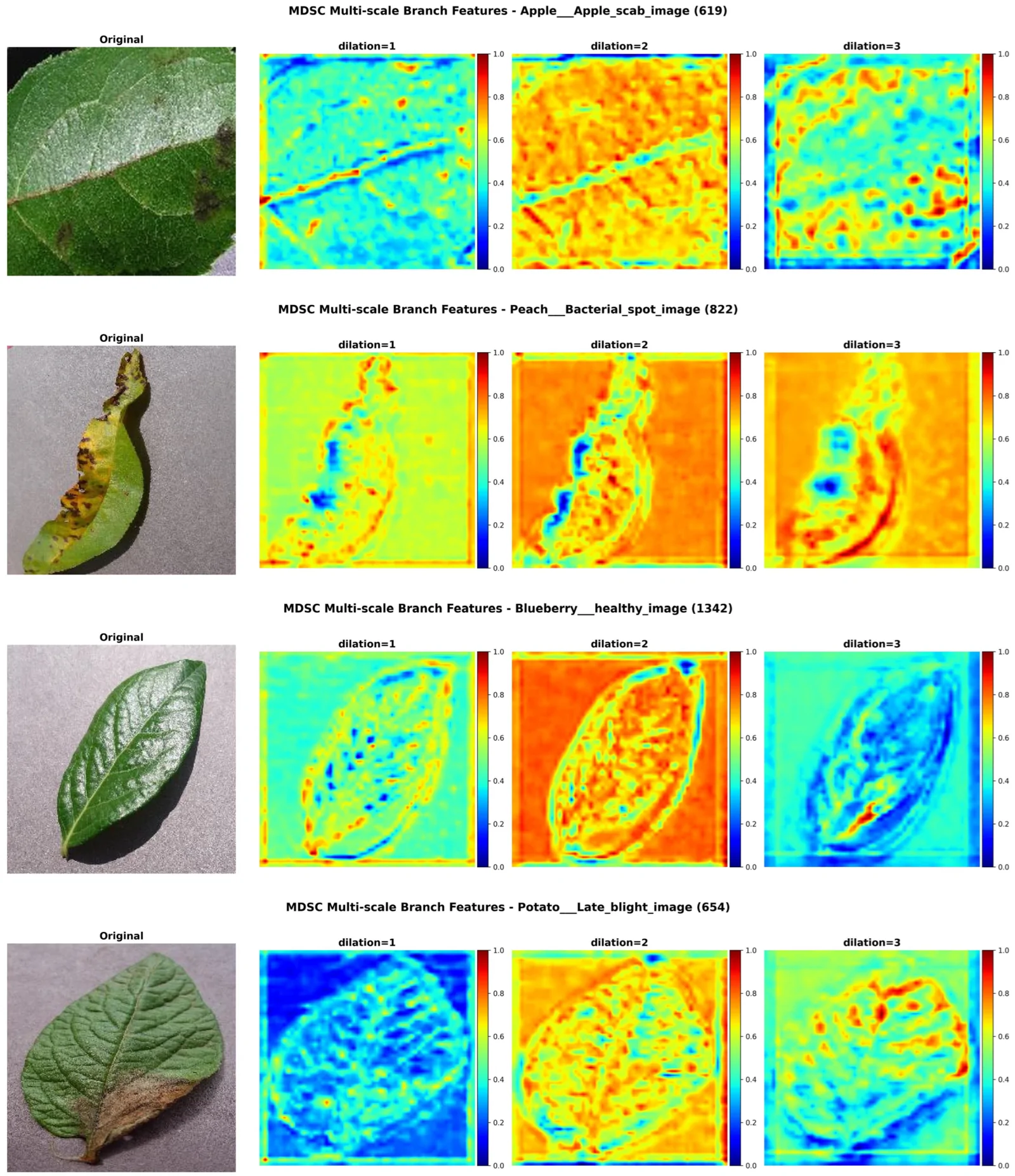
Multi-scale feature map visualization of the MDSC-Group branches (d = 1, 2, 3) and their fused output.

Multi-scale feature map visualization: to understand how the MDSC-Group captures lesions of varying sizes, we visualize the output feature maps of its three parallel depthwise separable convolution branches (dilation rates d = 1, 2, 3) in Figure 9. The branch with d = 1 predominantly activates on fine-grained lesion details, such as sharp necrotic spot edges and speckle patterns, which are critical for distinguishing early-stage symptoms. The branch with d = 2 responds to medium-sized symptomatic regions, capturing the transition zones between healthy and infected tissues. The branch with d = 3 produces the broadest activation coverage, encompassing large-area blights and coalesced lesions. The fused feature map, obtained via gradient-aware averaging, integrates these complementary scale-specific responses, producing a holistic representation that simultaneously preserves microscopic details and macroscopic disease patterns. This design ensures robust performance across lesion-scale variations without increasing computational overhead.

**Figure 10:**
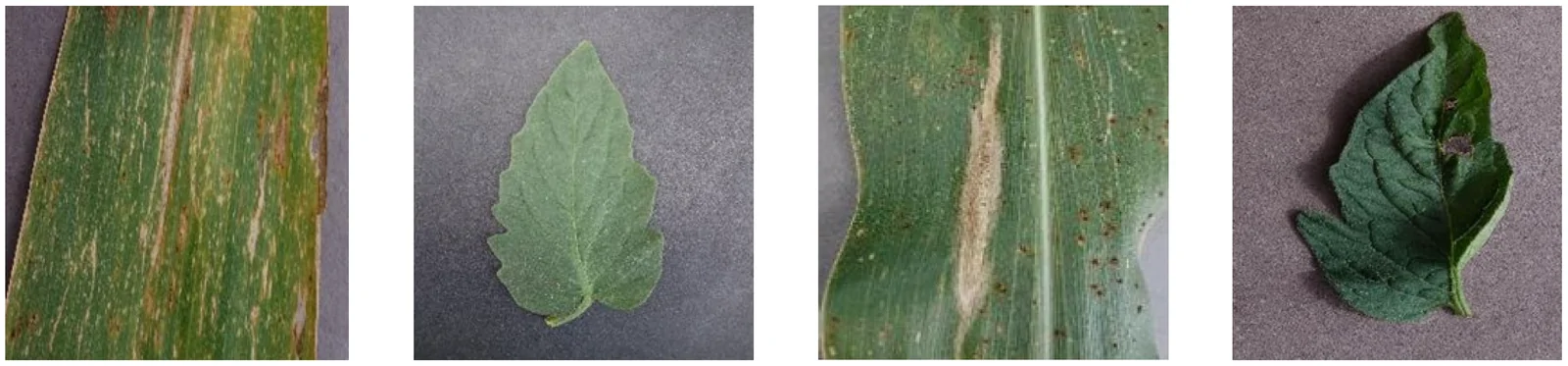
Representative misclassified samples from the PlantVillage dataset with failure mode analysis.

Analysis of misclassified samples: despite the high overall accuracy, we further inspect the failure cases to understand the remaining limitations of DPMFNet. Figure 10 presents representative misclassified samples from the PlantVillage test set. Although PlantVillage images are captured under controlled conditions with uniform backgrounds and consistent illumination, certain samples still pose significant challenges. Our analysis reveals that the majority of errors fall into three categories: (1) leaf curling or lesion overlapping, where symptomatic regions are partially hidden or deformed, making accurate localization difficult; (2) visually similar symptoms across different disease categories (e.g., different types of leaf spots or blights) that share nearly identical color, texture, and shape characteristics even under clear imaging conditions; and (3) extreme lesion sizes—either very small early-stage spots or large coalesced blights—that push the limits of scale-adaptive representation. Notably, many of these challenging samples would also be difficult for human experts to classify correctly based solely on visual inspection, suggesting that the remaining errors reflect inherent ambiguities in visual symptom classification rather than fundamental architectural deficiencies.

### 3.6. Ablation Study

To validate the contribution of each component and to examine the design choices within the SCDA module, we conduct comprehensive ablation studies on both the AI Challenger 2018 and PlantVillage datasets, using ResNet34 as the baseline. It should be noted that the results reported in Table 5 and Table 6 are obtained under a fixed random seed using the optimal weights for inference, without Bootstrap resampling, as these experiments focus on relative performance comparisons rather than statistical uncertainty estimation. All metrics are computed as macro-averages to ensure balanced evaluation across classes.

Internal ablation on pooling strategies within SCDA: to investigate the effectiveness of the dual-path pooling design in the channel attention branch, we evaluate three configurations on the AI Challenger 2018 dataset: (1) “both AvgPool,” where both parallel paths in the channel attention branch use Global Average Pooling; (2) “both MaxPool,” where both paths use Global Max Pooling; and (3) the complete SCDA design, which combines Global Average Pooling and Global Max Pooling in parallel (denoted as “SCDA only” in Table 5). As shown in Table 5, the “both AvgPool” configuration achieves 86.52% accuracy, while “both MaxPool” achieves 86.50% accuracy—both significantly outperforming the ResNet34 baseline (83.37%). However, the parallel combination of GAP and GMP demonstrates consistent and robust performance across both datasets, validating our design rationale that GAP preserves global symptom distribution while GMP captures extreme peak responses from small lesions. While minor numerical differences in individual metrics are observed under the single-run ablation setting, the overall cross-dataset consistency supports the effectiveness of the parallel fusion strategy.

Component-wise contribution analysis: Table 5 further demonstrates the individual and combined contributions of the two core modules. Integrating only the SCDA module improves accuracy from 83.37% to 86.23%, a gain of 2.86%, while increasing parameters marginally from 21.30M to 21.62M and FLOPs from 3.68G to 3.70G. Replacing standard convolutions with the MDSC-Group alone reduces model size significantly from 21.30M to 13.92M parameters and from 3.68G to 2.52G FLOPs, while still improving accuracy by 1.12% to 84.49%. When both modules are combined in the complete DPMFNet, the model achieves the best overall performance with 86.52% accuracy, 86.66% precision, 81.46% recall, and 86.37% F1-score, along with only 14.24M parameters and 2.55G FLOPs. These results confirm that SCDA and the MDSC-Group play complementary roles: SCDA enhances discriminative feature representation through precise spatial–channel reweighting, while MDSC-Group reduces computational cost and captures multi-scale contextual information. Their combination achieves a superior trade-off between accuracy and efficiency.

Cross-dataset generalization of ablation results: to verify that the observed improvements are not dataset-specific, we repeat the same ablation experiments on the PlantVillage dataset, as shown in Table 6. The performance patterns are highly consistent: SCDA-only improves accuracy from 99.07% to 99.64%, MDSC-only achieves 99.60% accuracy with significantly fewer parameters, and the complete DPMFNet reaches 99.72% accuracy with 99.72% precision, 99.66% recall, and 99.72% F1-score. The consistent improvement patterns across both datasets—one controlled and one field-based—strongly demonstrate the generalizability and robustness of each designed component, confirming that our architectural choices are not overfitted to a specific data distribution.

## 4. Discussion

### 4.1. Environmental and Agricultural Sustainability Benefits

Beyond technical metrics, the practical deployment of deep-learning-based disease prediction models like DPMFNet offers significant environmental and economic benefits for sustainable agriculture. Traditional disease management heavily relies on prophylactic or non-targeted broad-spectrum chemical applications, leading to excessive use of fungicides and pesticides. This over-application not only raises production costs for farmers but also accelerates environmental degradation, including soil contamination, runoff into aquatic ecosystems, and biodiversity loss.

By incorporating DPMFNet into smart agricultural platforms (such as edge-installed field monitors, autonomous spraying drones, or mobile diagnostic applications), farmers can achieve precise, early-stage disease-localized detection. Early identification allows for targeted spot-treatments rather than full-field chemical spraying, drastically reducing overall agrochemical inputs. Furthermore, accurate differentiation between pathogen types prevents the improper use of fungicides on non-fungal conditions (e.g., bacterial blights or nutrient deficiencies), mitigating the risk of pathogen resistance development. Consequently, lightweight models like DPMFNet serve as key technological enablers for Integrated Pest Management (IPM), bridging the gap between automated artificial intelligence and environmentally friendly, sustainable farming practices.

### 4.2. Limitations and Future Directions

Despite the strong performance demonstrated by DPMFNet on benchmark datasets, several limitations remain to be addressed in real-world agricultural deployments:

(1) Performance degradation under harsh environmental conditions: although the model exhibits high robustness on the AI Challenger 2018 field dataset, its recognition accuracy experiences noticeable degradation under extreme optical and structural occlusions. Specifically, strong backlighting creates severe contrast loss and flare artifacts, which mask subtle visual symptoms (such as faint chlorotic halos or early spot lesions). Additionally, heavy leaf overlapping and canopy shadowing impede the dynamic spatial receptive field of the SCDA module, occasionally leading to false negatives or misclassifications in dense crop stands.
(2) Future research directions: to mitigate these limitations and enhance field deployability, future work will focus on three key aspects:

Physics-informed and environment-adaptive data augmentation: integrating physical imaging models (e.g., direct light scattering and occlusion simulation algorithms) into the training pipeline to specifically improve network resilience against strong direct sunlight, backlighting, and foliage shadowing.

Multimodal and temporal feature fusion: combining visual imagery with localized microclimate sensor data (e.g., humidity, temperature, and canopy wetness) and multitemporal drone scans to resolve spatial ambiguity caused by leaf overlaps and canopy depth.

On-device edge optimization and field testing: building upon the favorable GPU-based efficiency results, we plan to quantize and compile DPMFNet via TensorRT/ONNX Runtime for deployment on resource-constrained edge hardware (e.g., NVIDIA Jetson modules or edge-drones), followed by extensive field validation across varying growth stages and outdoor light conditions.

## 5. Conclusions

Our DPMFNet model gets to 99.72% accuracy on PlantVillage and 86.52% on AI Challenger 2018—numbers that beat most standard baselines we tested under the same setup. The reason it works better is not just one trick, but two parts working together. There is the SCDA mechanism, which helps the network focus more precisely on actual lesion areas, even when those are small or partly hidden. And then there is the MDSC-Group, a block we designed to capture disease features at different scales without blowing up the model size. When you put them both into ResNet34, the whole thing ends up needing only 14.24 million parameters and usually settles down in training within about 70 epochs. That combination—higher accuracy, lower cost, faster convergence—is what gives the model its edge in both clean lab images and messy field photos.

Notably, while most existing models exhibit a substantial performance gap between the idealized PlantVillage and the complex real-world AI Challenger 2018 datasets, our method maintains consistently high performance across both domains, highlighting its effectiveness on both controlled and field-captured disease images.

Future work will explore the extension of this architecture to open-environment imagery and multi-crop joint diagnosis scenarios, further advancing its applicability in real-world precision agriculture systems.

## Figures and Tables

**Table 1:** Network architecture configuration of DPMFNet.

Stage	Output Resolution	Number of Channels	Block Type	SCDA Enabled
conv1	112 × 112	64	Conv + BN + ReLU + MaxPool	No
conv2_x	56 × 56	64	AttMDSCBlock × 3	No
conv3_x	28 × 28	128	AttMDSCBlock × 4	Yes
conv4_x	14 × 14	256	AttMDSCBlock × 6	Yes
conv5_x	7 × 7	512	AttMDSCBlock × 3	Yes

**Table 2:** Experimental results of various models on the AI Challenger 2018 dataset.

Model	Accuracy	Precision	Recall	F1_Score
AlexNet	81.22 ± 0.58	81.75 ± 0.57	81.22 ± 0.58	81.00 ± 0.59
VGG16	81.69 ± 0.57	82.13 ± 0.57	81.69 ± 0.57	81.41 ± 0.59
Densnet121	84.45 ± 0.55	84.81 ± 0.53	84.45 ± 0.55	84.30 ± 0.55
mobilenet_v2	84.57 ± 0.54	85.18 ± 0.52	84.57 ± 0.54	84.35 ± 0.55
resnet18	82.56 ± 0.57	84.17 ± 0.59	82.56 ± 0.57	81.89 ± 0.61
resnet34	83.36 ± 0.57	84.23 ± 0.57	83.36 ± 0.57	83.07 ± 0.59
resnet50	82.74 ± 0.56	83.64 ± 0.55	82.74 ± 0.56	81.98 ± 0.60
resnet101	83.86 ± 0.55	83.99 ± 0.56	83.86 ± 0.55	83.70 ± 0.57
resnet152	82.82 ± 0.55	83.46 ± 0.54	82.82 ± 0.55	82.79 ± 0.55
efficientnet-b0	83.26 ± 0.54	83.93 ± 0.55	83.26 ± 0.54	83.05 ± 0.56
googlenet	84.77 ± 0.53	85.57 ± 0.51	84.77 ± 0.53	84.38 ± 0.55
shufflenet_v2_x0_5	84.38 ± 0.54	84.80 ± 0.55	84.38 ± 0.54	84.21 ± 0.55
shufflenet_v2_x1_0	85.28 ± 0.51	85.70 ± 0.50	85.28 ± 0.51	85.13 ± 0.52
convnext-t	84.91 ± 0.54	85.46 ± 0.53	84.91 ± 0.54	84.68 ± 0.55
convnext-s	84.20 ± 0.54	85.48 ± 0.51	84.20 ± 0.54	84.21 ± 0.54
convnext-b	84.51 ± 0.54	85.25 ± 0.56	84.51 ± 0.54	83.82 ± 0.58
convnext-l	84.78 ± 0.54	85.66 ± 0.53	84.78 ± 0.54	84.70 ± 0.55
RepViT [36]	85.77 ± 0.50	86.12 ± 0.50	85.77 ± 0.50	85.65 ± 0.51
fastvit_t8 [37]	69.73 ± 0.70	75.87 ± 0.73	69.73 ± 0.70	68.29 ± 0.75
DPMFNet	86.48 ± 0.50	86.63 ± 0.50	86.48 ± 0.50	86.33 ± 0.51

**Table 3:** Experimental results of various models on the PlantVillage dataset.

Model	Accuracy	Precision	Recall	F1_Score
AlexNet	98.69 ± 0.15	98.70 ± 0.15	98.69 ± 0.15	98.69 ± 0.15
VGG16	98.63 ± 0.15	98.65 ± 0.15	98.63 ± 0.15	98.63 ± 0.15
Densenet121	99.67 ± 0.07	99.68 ± 0.07	99.67 ± 0.07	99.67 ± 0.07
mobilenet_v2	99.60 ± 0.08	99.61 ± 0.08	99.60 ± 0.08	99.60 ± 0.08
resnet18	99.04 ± 0.13	99.08 ± 0.12	99.04 ± 0.13	99.03 ± 0.13
resnet34	99.08 ± 0.12	99.10 ± 0.12	99.08 ± 0.12	99.08 ± 0.12
resnet50	99.32 ± 0.10	99.34 ± 0.10	99.32 ± 0.10	99.31 ± 0.10
resnet101	99.14 ± 0.12	99.18 ± 0.11	99.14 ± 0.12	99.14 ± 0.12
resnet152	99.08 ± 0.12	99.09 ± 0.12	99.08 ± 0.12	99.08 ± 0.12
efficientnet-b0	99.49 ± 0.09	99.50 ± 0.09	99.49 ± 0.09	99.49 ± 0.09
googlenet	99.48 ± 0.10	99.48 ± 0.09	99.48 ± 0.10	99.48 ± 0.10
shufflenet_v2_x0_5	99.24 ± 0.11	99.26 ± 0.11	99.24 ± 0.11	99.24 ± 0.11
shufflenet_v2_x1_0	99.47 ± 0.09	99.48 ± 0.09	99.47 ± 0.09	99.47 ± 0.09
convnext-t	99.42 ± 0.10	99.44 ± 0.10	99.42 ± 0.10	99.42 ± 0.10
convnext-s	99.23 ± 0.11	99.25 ± 0.11	99.23 ± 0.11	99.23 ± 0.12
convnext-b	99.52 ± 0.08	99.53 ± 0.08	99.52 ± 0.08	99.52 ± 0.09
convnext-l	99.34 ± 0.10	99.36 ± 0.10	99.34 ± 0.10	99.34 ± 0.10
RepViT	99.01 ± 0.13	99.02 ± 0.13	99.01 ± 0.13	99.01 ± 0.13
fastvit_t8	96.44 ± 0.24	96.46 ± 0.24	96.44 ± 0.24	96.42 ± 0.24
DPMFNet	99.72 ± 0.07	99.72 ± 0.07	99.72 ± 0.07	99.72 ± 0.07

**Table 4:** Model complexity comparison.

Model	Params (M)	FLOPs (G)	Latency (ms)	FPS	Peak GPU Memory (M)
alexnet	57.16	0.71	0.13	7572.94	381.67
vgg16	134.42	15.47	0.69	1441.78	2948.29
densenet121	6.99	2.9	0.69	1459.00	1336.34
mobilenet_v2	2.27	0.33	0.23	4268.08	1365.14
resnet18	11.2	1.82	0.20	5043.37	909.46
resnet34	21.3	3.68	0.35	2878.16	948.06
resnet50	23.59	4.13	0.61	1627.78	1446.54
resnet101	42.58	7.86	1.01	987.22	1518.72
resnet152	58.22	11.6	1.45	689.13	1578.59
efficientnet_b0	4.06	0.41	0.29	3437.62	1372.88
googlenet	5.64	1.51	0.26	3776.11	961.99
shufflenet_v2_x0_5	0.38	0.04	0.05	18,870.52	379.82
shufflenet_v2_x1_0	1.29	0.15	0.11	9363.95	383.31
convnext-t	27.84	4.46	0.86	1156.11	1660.97
convnext-s	49.47	8.7	1.48	675.15	1743.50
convnext-b	87.58	15.37	2.26	442.68	2375.93
convnext-l	196.26	34.39	4.47	223.49	3771.08
RepViT	4.75	0.85	0.32	3171.11	619.20
fastvit_t8	3.26	0.53	0.36	2792.00	1270.00
DPMFNet	14.24	2.55	0.74	1350.03	921.45

**Table 5:** Ablation study results on AI Challenger 2018 dataset.

Model	Params (M)	FLOPs (G)	Accuracy	Precision	Recall	F1_Score
resnet34	21.30	3.68	83.37	84.21	77.81	83.08
+scda only	21.62	3.70	86.23	86.40	81.11	86.14
+scda only (both Avgpool)	21.62	3.70	86.52	86.53	81.46	86.36
+scda only (both Maxpool)	21.62	3.70	86.50	86.50	81.58	86.38
+mdsc only	13.92	2.52	84.49	85.59	79.73	84.20
DPMFNet	14.24	2.55	86.52	86.66	81.46	86.37

**Table 6:** Ablation study results on PlantVillage dataset.

Model	Params (M)	FLOPs (G)	Accuracy	Precision	Recall	F1_Score
resnet34	21.30	3.68	99.07	99.09	98.92	99.07
+scda only	21.62	3.70	99.64	99.64	99.51	99.64
+scda only (both Avgpool)	21.62	3.70	99.49	99.49	99.34	99.49
+scda only (both Maxpool)	21.62	3.70	99.45	99.46	99.34	99.45
+mdsc only	13.92	2.52	99.60	99.61	99.48	99.60
DPMFNet	14.24	2.55	99.72	99.72	99.66	99.72

## Data Availability

The experiments were conducted using two publicly available datasets: (1) The AI Challenger 2018 Crop Disease Classification Dataset, which is accessible via the AI Studio platform at https://aistudio.baidu.com/datasetdetail/76075 (accessed on 28 June 2026); (2) The PlantVillage Plant Disease Dataset, available on Kaggle at https://www.kaggle.com/datasets/emmarex/plantdisease/data (accessed on 28 June 2026).

## References

[1] Savary S., Willocquet L., Pethybridge S.J., Esker P., McRoberts N., Nelson A. (2019). The global burden of pathogens and pests on major food crops. Nat. Ecol. Evol..

[2] Li L., Zhang S., Wang B. (2021). Plant disease detection and classification by deep learning—A review. IEEE Access.

[3] Zhang Q., Zhu Y., Cordeiro F.R., Chen Q. (2025). PSSCL: A progressive sample selection framework with contrastive loss designed for noisy labels. Pattern Recognit..

[4] Jin G., Zhang Q., Cheng Y., Xu M., Zhu Y., Yu D., Yuan Y., Li J., Shi J. (2026). Enhancing feature discrimination with pseudo-labels for foundation model in segmentation of 3D medical images. Neural Netw..

[5] Yuan Y., Cheng Y., Pan B., Jin G., Yu D., Ye M., Zhang Q. (2025). A Multi-Modal Attention Fusion Framework for Road Connectivity Enhancement in Remote Sensing Imagery. Mathematics.

[6] Krizhevsky A., Sutskever I., Hinton G.E. (2017). Imagenet classification with deep convolutional neural networks. Commun. ACM.

[7] Simonyan K., Zisserman A. (2014). Very deep convolutional networks for large-scale image recognition. arXiv.

[8] He K., Zhang X., Ren S., Sun J. (2016). Deep residual learning for image recognition. Proceedings of the IEEE Conference on Computer Vision and Pattern Recognition.

[9] Mohanty S.P., Hughes D.P., Salathé M. (2016). Using deep learning for image-based plant disease detection. Front. Plant Sci..

[10] Khan A.I., Quadri S.M.K., Banday S., Shah J.L. (2022). Deep diagnosis: A real-time apple leaf disease detection system based on deep learning. Comput. Electron. Agric..

[11] Zeng W., Li H., Hu G., Liang D. (2022). Lightweight dense-scale network (LDSNet) for corn leaf disease identification. Comput. Electron. Agric..

[12] Hu J., Shen L., Sun G. (2018). Squeeze-and-excitation networks. Proceedings of the IEEE Conference on Computer Vision and Pattern Recognition.

[13] Woo S., Park J., Lee J.-Y., Kweon I.S. (2018). Cbam: Convolutional block attention module. Proceedings of the European Conference on Computer Vision (ECCV).

[14] Hou Q., Zhou D., Feng J. (2021). Coordinate attention for efficient mobile network design. Proceedings of the IEEE/CVF Conference on Computer Vision and Pattern Recognition.

[15] Chen Y., Huang Y., Zhang Z., Wang Z., Liu B., Liu C., Huang C., Dong S., Pu X., Wan F. (2023). Plant image recognition with deep learning: A review. Comput. Electron. Agric..

[16] Lin T.-Y., Dollár P., Girshick R., He K., Hariharan B., Belongie S. (2017). Feature pyramid networks for object detection. Proceedings of the IEEE Conference on Computer Vision and Pattern Recognition.

[17] Chen L.-C., Papandreou G., Schroff F., Adam H. (2017). Rethinking atrous convolution for semantic image segmentation. arXiv.

[18] Szegedy C., Liu W., Jia Y., Sermanet P., Reed S., Anguelov D., Erhan D., Vanhoucke V., Rabinovich A. (2015). Going deeper with convolutions. Proceedings of the IEEE Conference on Computer Vision and Pattern Recognition.

[19] Xiao D., Zeng R., Liu Y., Huang Y., Liu J., Feng J., Zhang X. (2022). Citrus greening disease recognition algorithm based on classification network using TRL-GAN. Comput. Electron. Agric..

[20] Zhu J.-Y., Park T., Isola P., Efros A.A. (2017). Unpaired image-to-image translation using cycle-consistent adversarial networks. Proceedings of the IEEE International Conference on Computer Vision.

[21] Duhan S., Gulia P., Gill N.S., Aziz A.L., Aljuaid M., Banshal S.K., Bansal R. (2025). LiteCShuffle: A lightweight and efficient deep learning approach for plant disease classification. Cogent Food Agric..

[22] Hong W., Ling S., Zhu P., Wang Z., Zhao R., Liu Y., Dong M. (2026). Lightweight Vision–Transformer Network for Early Insect Pest Identification in Greenhouse Agricultural Environments. Insects.

[23] Chen Y., Wang A., Liu Z., Yue J., Zhang E., Li F., Zhang N. (2025). MoSViT: A lightweight vision transformer framework for efficient disease detection via precision attention mechanism. Front. Artif. Intell..

[24] Sun W., Xu Z., Xu K., Ru L., Yang R., Wang R., Xing J. (2025). Ultra-lightweight tomatoes disease recognition method based on efficient attention mechanism in complex environment. Front. Plant Sci..

[25] Brahimi M., Boukhalfa K., Moussaoui A. (2017). Deep learning for tomato diseases: Classification and symptoms visualization. Appl. Artif. Intell..

[26] Ebrahimi M.A., Khoshtaghaza M.H., Minaei S., Jamshidi B. (2017). Vision-based pest detection based on SVM classification method. Comput. Electron. Agric..

[27] Wen C., Guyer D. (2012). Image-based orchard insect automated identification and classification method. Comput. Electron. Agric..

[28] Wen C., Guyer D.E., Li W. (2009). Local feature-based identification and classification for orchard insects. Biosyst. Eng..

[29] Zeng W., Li M. (2020). Crop leaf disease recognition based on Self-Attention convolutional neural network. Comput. Electron. Agric..

[30] Chen J., Zhang D., Zeb A., Nanehkaran Y.A. (2021). Identification of rice plant diseases using lightweight attention networks. Expert Syst. Appl..

[31] Kamal K.C., Yin Z., Wu M., Wu Z. (2019). Depthwise separable convolution architectures for plant disease classification. Comput. Electron. Agric..

[32] Waheed A., Goyal M., Gupta D., Khanna A., Hassanien A.E., Pandey H.M. (2020). An optimized dense convolutional neural network model for disease recognition and classification in corn leaf. Comput. Electron. Agric..

[33] Zhang S., Zhang S., Zhang C., Wang X., Shi Y. (2019). Cucumber leaf disease identification with global pooling dilated convolutional neural network. Comput. Electron. Agric..

[34] Wang L., Sun J., Wu X., Shen J., Lu B., Tan W. (2020). Identification of crop diseases using improved convolutional neural networks. IET Comput. Vis..

[35] Li E., Wang L., Xie Q., Gao R., Su Z., Li Y. (2023). A novel deep learning method for maize disease identification based on small sample-size and complex background datasets. Ecol. Inform..

[36] Wang A., Chen H., Lin Z., Han J., Ding G. (2024). Rep ViT: Revisiting Mobile CNN from ViT Perspective. Proceedings of the 2024 IEEE/CVF Conference on Computer Vision and Pattern Recognition (CVPR), Seattle, WA, USA, 16–22 June 2024.

[37] Anasosalu Vasu P.K., Gabriel J., Zhu J.J., Tuzel O., Ranjan A. (2023). FastViT: A Fast Hybrid Vision Transformer using Structural Reparameterization. Proceedings of the 2023 IEEE/CVF International Conference on Computer Vision (ICCV), Paris, France, 1–6 October 2023.

